# Synthesis, Characterization and Evaluation of Supercapacitive Response of Dodecylbenzenesulphonic Acid (DBSA) Doped Polypyrrole/Zirconium Dioxide Composites

**DOI:** 10.3390/polym13224035

**Published:** 2021-11-22

**Authors:** Rizwan Ullah, Mehtab Khan, Rozina Khattak, Nadia Khan, Muhammad Sufaid Khan, Yaser A. El-Badry

**Affiliations:** 1 National Center of Excellence in Physical Chemistry, University of Peshawar, Peshawar 25120, Pakistan; mehtabk324@uop.edu.pk (M.K.); Khannadia@uop.edu.pk (N.K.); 2 Department of Chemistry, Shaheed Benazir Bhutto Women University, Peshawar 25000, Pakistan; 3 Department of Chemistry, University of Malakand, Chakdara 18800, Pakistan; sufaidkhan1984@uom.edu.pk; 4 Department of Chemistry, Faculty of Science, Taif University, Khurma, P.O. Box 11099, Taif 21944, Saudi Arabia; y.elbadry@tu.edu.sa

**Keywords:** polypyrrole, zirconium dioxide, composites, supercapacitor

## Abstract

An in-situ chemical oxidative method was used to effectively synthesize a promising supercapacitor material based on PPy/ZrO_2_ composites. The synthesized materials were characterized by different analytical techniques, such as UV/visible (UV/Vis) spectroscopy, Fourier-transform infra-red spectroscopy (FTIR), X-ray diffraction (XRD), thermogravimetric analysis (TGA), and scanning electron microscopy (SEM). The inclusion of ZrO_2_ into the PPy matrix was verified by vibrational spectra and structural analyses. The (TGA) results showed that incorporating ZrO_2_ into the polymeric matrix improved its thermal stability. In addition, the electrochemical properties of the synthesizedmaterials were investigated byusing cyclic voltammetry (CV) and galvanostatic charge/discharge (GCD). The PPy/ZrO_2_ composite demonstrated excellent super capacitive performance, and high specific capacity of 337.83 F/g, with an exceedingly high energy density of 187.68 Wh/kg at a power density of 1000 W/kg. The composite materials maintain good stability after 1000 charge and discharge cycles, with 85% capacitance retention. The PPy/ZrO_2_ possesses a high capacitance, an attractive micro-morphology, and a simple synthesis method. The findings indicate that the PPy/ZrO_2_ composite could be a promising electrode material for high-performance supercapacitor applications.

## 1. Introduction

Electrochemical capacitors, also known as supercapacitors, are known for their ideal power storage capacity, which is achieved through rapid/reversible redox reactions and/or phase-change processes on the surface or subsurface areas of modified electrodes in various types of portable electronic equipment [1,2,3]. Supercapacitors show a number of interesting characteristics, including fast charge/discharge rate, safe operational features, ideal cyclic stability, and highpower density [4,5]. Solid-state supercapacitors demonstrate advantages such as ideal safety, light weight, and high flexibility, as compared to that of liquid electrolyte-based supercapacitors, and they are essential for the development of state-of-the-artportable electronic devices [6,7,8]. Supercapacitors occupied a very large significant portion of the market and research area among many electrochemical energy storage devices, due to their high power density, which can store significant amount of energy, long life cyclic stability (100 to 1000 of charge/discharge cycles before any significant deterioration of the charge capacity), andalmost unlimited charge/discharge rate [9].

The conducting polymer Polypyrrole (PPy) is broadly considered as a perfect candidate for the development of both electric double-layer capacitors (EDLC) and pseudocapacitors due to its fast and reversible redox process caused by the pi-conjugated polymeric chain [10,11]. Due to its advantages over its equivalents among the numerous conducting polymers (CPs), PPy performs admirably as an electrode material for the creation of supercapacitors. These noteworthy advantages of PPy include promising mechanical strength, ideal specific capacity, high electrical conductivity, and biocompatibility [12,13,14]. Still, like other CPs, PPy has a number of drawbacks, such as volumetric shrinking during discharge and a decrease in cyclic stability, which could be mitigated by adding potential modifiers [15,16]. Because of their rapid redox kinetics and perfect capacitance, transition metal oxides were widely employed to improve the PPy’s cyclic stability [17,18]. Different researchers synthesized nanocomposites of PPy with numerous MOs, including TiO_2_ [19], Fe_2_O_3_ [20], SiO_2_ [21], ZnO [22] and CeO_2_ [23], due to the large potential of CPs and polymers doped with inorganic metal oxides (MOs), which increase the performance of materials in many applications. High electron affinity, better mechanical characteristics, and high electrical conductivity may all be achieved by doping MOs in CPs [24]. These nanomaterial-doped CPs are employed for a wide range of applications and purposes. For supercapacitors, high-performance ordered porous Polypyrrole/ZnO sheets with enhanced specific capacitance were developed [25]. PPy and its composites with titanium oxide demonstrate increased supercapacitative qualities [26]. Polypyrrole@silica composites with good performance as electrode materials for Lithium-ion batteries were prepared using a solution polymerization process [27]. The development of new materials with encouraging electrochemical properties is an important goal for energy storage applications. Furthermore, low working potential, low energy density, and large self-charge/discharge currents are all issues that must be addressed in the development of next-generation supercapacitors. Supercapacitors can use MOs electrodes, although they have limited current capability and poor cycle stability. As a result, there is a compelling need to enhance the electrochemical characteristics of MOs and CPs [28].

In the current study, PPy/ZrO_2_ composites were synthesized by an in-situ chemical oxidative polymerization method with improved electrochemical performance and specific capacitance at a scan rate of 100 mV s^−1^. The solid-state supercapacitor device, which is made up of as-prepared PPy/ZrO_2_ composite electrodes, has good specific capacitance of 337.83 F/g and a maximum energy density of 187.68 Wh/kg. These findings indicate that the PPy/ZrO_2_ composites could be promising electrode materials for high-performance supercapacitor applications, which was not previously reported.

## 2. Materials and Methods

### 2.1. Materials

The reagent grade pyrrole monomer (Fluka) was distilled twice before use. The following chemicals were used as received: dodecyl-benzenesulphonic acid (DBSA), chloroform, ammonium persulphate (APS), acetone and N-methyl-2-pyrrolidone (NMP) were purchased from Sigma–Aldrich (St. Louis, MO, USA). However, methanol and sulfuric acid were provided by Scharlau (Sentmenat, Spain). Double-distilled water was used for solution preparation and glassware washing/rinsing. The compounds that were utilized are listed in Table 1 below.

### 2.2. Synthesis of Polypyrrole (PPy)

In a round bottom flask placed in an ice bath, 0.2 mL pyrrole monomer and 50 mL deionized water were added and stirred to make PPy. After 10 min, 3.5 mL dodecyl-benzenesulfonic acid (DBSA) was added and stirred for another 10 min, then 10 mL ammonium per sulphate (APS) solution (0.1 M) was added dropwise as an initiator. The reaction mixture was stirred for 24 h at 5 to 10 °C. The precipitate was separated by centrifugation (7000 rpm) after 24 h and washed several times with water followed by acetone. The resulting black powder was vacuum-dried at 60 °C for 12 h and labelled as PPy.

### 2.3. Synthesis of Polypyrrole/Zirconium Dioxide (PPy/ ZrO_2_) Composites

The same procedure was followed for the synthesis of PPy/ZrO_2_ composites by adding different amounts (0.1 g, 0.15 g, 0.2 g, 0.25 g and 0.3 g) of ZrO_2_ into the reaction mixture just after the addition of DBSA. The samples thus obtained were named as PPy/ZrO_2_ 1, PPy/ZrO_2_ 2, PPy/ZrO_2_ 3, PPy/ZrO_2_ 4, and PPy/ZrO_2_ 5, respectively.

### 2.4. Apparatus

For electrochemical measurements Autolab, and three electrode cell having different electrodes such as glassy carbon as working electrode, gold as counter electrode, and silver-silver chloride as reference electrode, were used.

## 3. Results and Discussion

### 3.1. UV-Visible Characterization

**Figure 1:**
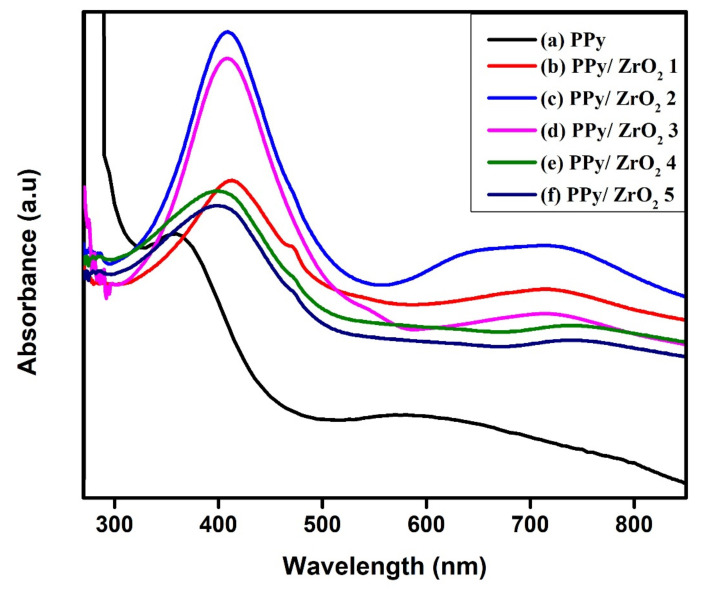
UV-Visible spectra of (**a**) PPy, (**b**) PPy/ZrO_2_ 1, (**c**) PPy/ZrO_2_ 2, (**d**) PPy/ZrO_2_ 3, (**e**) PPy/ZrO_2_ 4, (**f**) PPy/ZrO_2_ 5.

The UV-visible spectra of pure PPy and its ZrO_2_ composites were measured in the 200–900 nm region (Figure 1a–f). Pure PPy spectra reveal two distinct wide absorption bands, located at 359 nm and 589 nm, respectively (Figure 1a). The π-π* transition is responsible for the initial absorption band. At 584 nm, the second wide absorption band suggests that the PPy is in oxidized and non-conducting state [29]. Figure 1b–f shows the absorption spectra of PPy/ZrO_2_ composites. The composites also show two absorption peaks, with a peak in the region of 401–410 nm indicating the PPy ring’s π-π* transition. The second absorption peak above 700 nm in the composites belongs to the sum of polarons and bipolarons [30]. The broad peaks for PPy/ZrO_2_ composites curves (b), (c), (d), (e) and (f) show that when the amount of ZrO_2_ in the composites rises, the peak for the sum of polaron and bipolaron shifts towards the higher wavelength [30]. This results in a narrowing of the band gap and an increase in the composites’ conductivity.

### 3.2. UV Band Gap Calculation

The optical band gap was computed using the Tauc Equation (1). The UV-visible spectra of PPy and its composites aid in the identification of their optical band gaps, where αand *h* indicate the absorption coefficients and Planck’s constant, respectively. The symbols *ν*, *A*, and *Eg* stand for light frequency, a constant, and band gap, respectively.

(1) $${\left(\alpha h\nu \;\right)}^{2}=A{\left(h\nu -Eg\right)}^{n}$$

**Figure 2:**
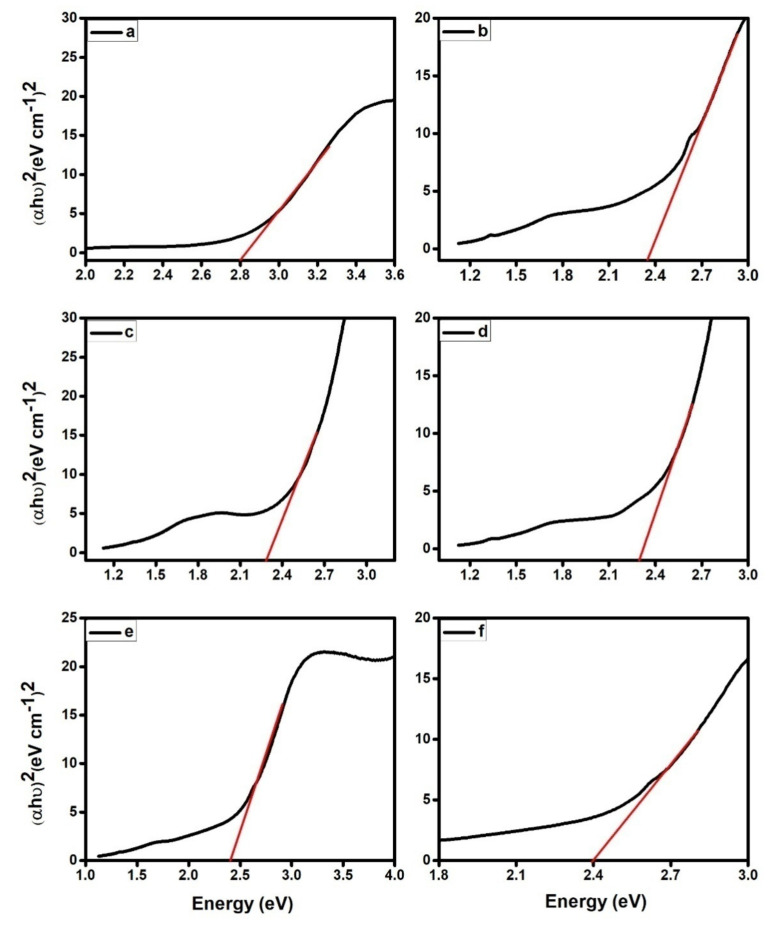
Band gap calculation for (**a**) PPy, (**b**) PPy/ZrO_2_ 1, (**c**) PPy/ZrO_2_ 2, (**d**) PPy/ZrO_2_ 3, (**e**) PPy/ZrO_2_ 4, (**f**) PPy/ZrO_2_ 5.

PPy and PPy/ZrO_2_ 1, PPy/ZrO_2_ 2, PPy/ZrO_2_ 3, PPy/ZrO_2_ 4, and PPy/ZrO_2_ 5 had band gaps of 2.8, 2.36, 2.28, 2.29, 2.4, and 2.39, respectively, as shown in Figure 2. These findings reveal that increasing the quantity of ZrO_2_ in the polymer matrix reduces the band gap, resulting in an increase in conductivity. As demonstrated in Figure 2, the band gap of PPy/ZrO_2_ 2 is less than that of other composites and pure PPy.

### 3.3. Vibrational Spectroscopy/FTIR Analysis

**Figure 3:**
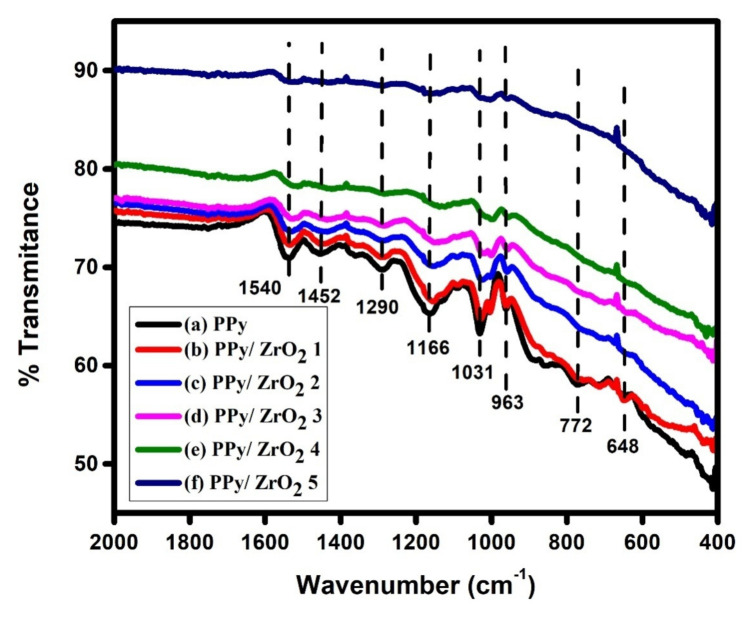
FTIR spectra (**a**) PPy, (**b**) PPy/ZrO_2_ 1, (**c**) PPy/ZrO_2_ 2, (**d**) PPy/ZrO_2_ 3, (**e**) PPy/ZrO_2_ 4, (**f**) PPy/ZrO_2_ 5.

FTIR spectra of PPy and PPy/ZrO_2_ composites are shown in Figure 3. The C–C stretching vibration in the pyrrole ring shows up as an absorption band at 1540 cm^−1^ in PPy. The ring’s C–N stretching vibration is represented by the band at 1452 cm^−1^ in the PPy spectrum [21]. The C–N in-plane band is at 1290 cm^−1^, and the C–H bending modes are at 1164 cm^−1^ and 1031 cm^−1^ [21,22]. The C–C and C–H out-of-plane deformation vibration bands are at 963 and 891 cm^−1^, respectively [21,22]. Except for the C–C, C–N and C–H bending modes of the pyrrole ring, the PPy/ZrO_2_ composite spectra generated with various ZrO_2_ weights displayed superimposed absorption bands of both components [21]. For pure PPy, the bands at 1540 cm^−1^correspond to C–C stretching vibration, and 1452 cm^−1^ to C–N stretching vibration, and 1031 cm^−1^ to C–H bending modes are red-shifted to 1516, 1431, and 1000cm^−1^ for the PPy/ZrO_2_ 5 composite, respectively. The small absorption band at 963 cm^−1^ was slightly red shifted down to 956 cm^−1^. The peak intensity of pure PPy declines as the concentration of ZrO_2_ grows and for the PPy/ZrO_2_ 5 composite, it virtually vanishes (Figure 3). The formation of PPy/ZrO_2_ composites is indicated by these findings.

### 3.4. X-ray Diffraction Studies

**Figure 4:**
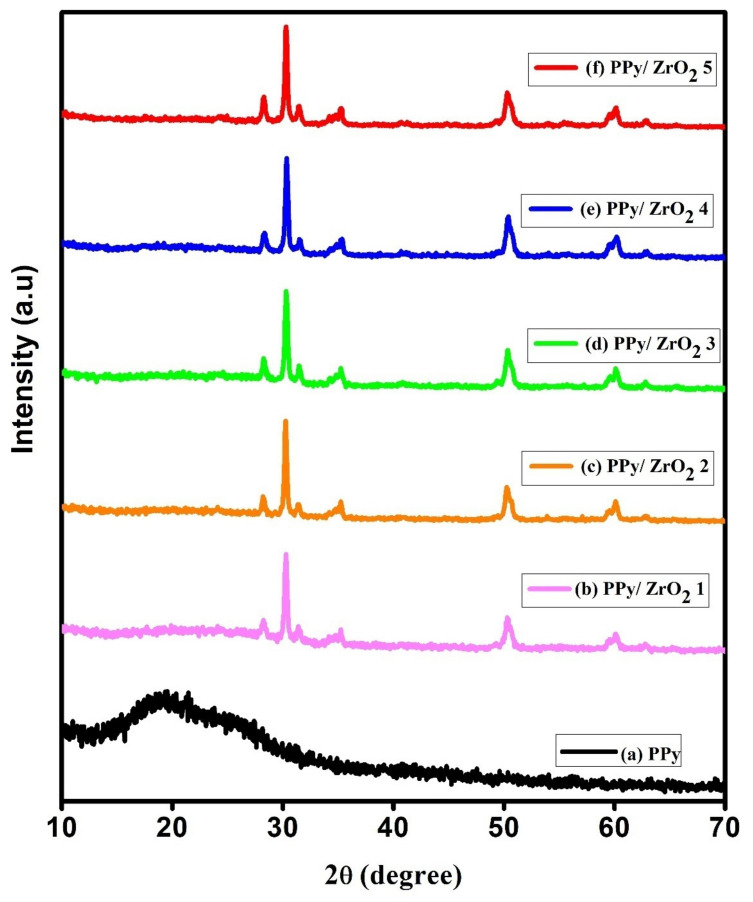
XRD spectra (**a**) PPy, (**b**) PPy/ZrO_2_ 1, (**c**) PPy/ZrO_2_ 2, (**d**) PPy/ZrO_2_ 3, (**e**) PPy/ZrO_2_ 4, (**f**) PPy/ZrO_2_ 5.

The X-ray diffraction patterns of PPy and PPy/ZrO_2_ composites are shown in Figure 4. Only PPy has a broad peak at 2θ = 12° to 30°, with tiny shoulders at 17°, 19°, and 21° in the sample. In the PPy sample, the shoulder at 2θ = 17° and 2θ = 19° reflects the distance between the benzene ring planes in adjacent PPy chains. The PPy, like other CPs, has a broad peak, which is thought to imply a semicrystalline nature [31,32].

The composites’ XRD diffractograms exhibit a prominent and intense peak at 2θ = 30°, as well as less intense peaks at 2θ = 35°, 50°, and 60°, all of which may be attributed to ZrO_2_ inside the polymer matrix [33]. These peaks for ZrO_2_ in the PPy matrix imply an increase in crystallinity, which is consistent with the band gap calculation and related increase in conductivity. The broad PPy peak is flattened by ZrO_2_ inclusion, and the ZrO_2_ peaks dominate the diffractograms. It may be deduced from this that the addition of ZrO_2_ had a significant impact on the crystallinity of PPy [34]. Scherrer’s Equation (2) was used to compute the average crystallite size.

(2) $$D=\frac{k\lambda }{\beta Cos\theta }$$

where *β* is the full width at half maximum (FWHM), *λ* is the X-ray wavelength, θ is the diffraction angle, and *k* is the Scherrer’s constant of order one. The average crystallite size in PPy/ZrO_2_ composites is 19.56 nm.

### 3.5. Thermogravimetric Analysis (TGA)

**Figure 5:**
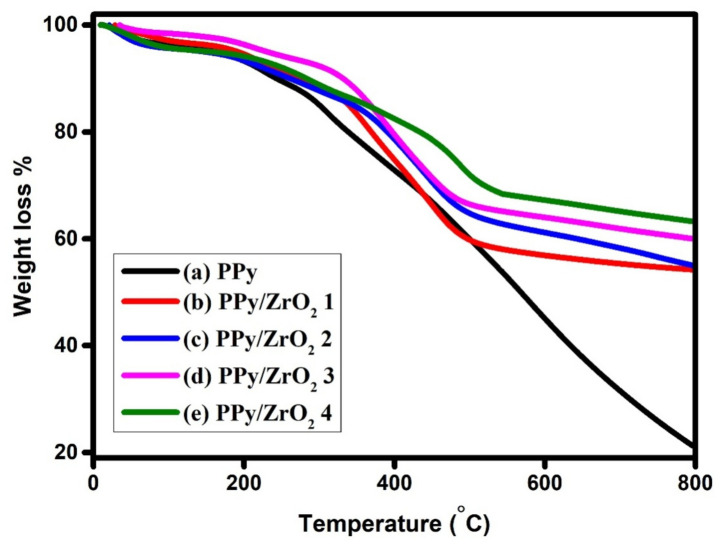
TGA curves of (**a**) PPy (**b**) PPy/ZrO_2_ 1 (**c)** PPy/ZrO_2_ 2 (**d**) PPy/ZrO_2_ 3 (**e**) PPy/ZrO_2_ 4.

Figure 5 shows the thermograms of PPy and PPy/ZrO_2_ composites, which demonstrate three step weight losses. For pure PPy, the absorbed water molecules are evaporated in the first stage, resulting in a weight loss of 5% at temperatures ranging from 25 °C to 150 °C. The DBSA molecules are removed from the polymer matrix in the second stage. The decomposition of the main polymer chain occurs in the third stage at high temperatures ranging from 425 °C to 800 °C, with 21.29% weight retention. In composites, ZrO_2_ plays a crucial function in modifying the composites’ thermal behavior. The addition of ZrO_2_ to PPy/ZrO_2_ composites improves thermal stability significantly, as evidenced by curves (b–e) in Figure 5 [32]. The strong association/interaction between PPy and ZrO_2_ particles-particles is responsible for the improved thermal stability [35]. In comparison to pure PPy, the major decomposition in composites begins at high temperatures, resulting in a 63.27% increase in weight retention. Table 2 summarizes weight loss and weight retention at different temperatures.

### 3.6. Morphological Study

**Figure 6:**
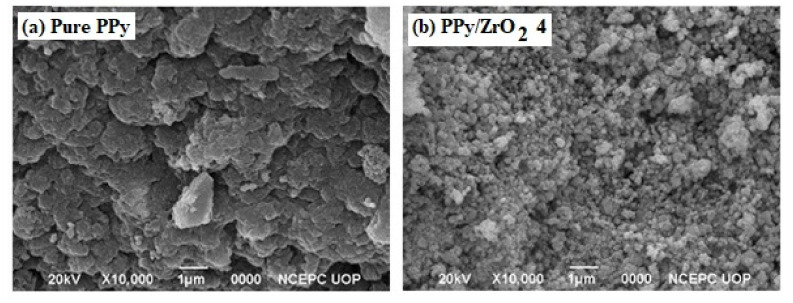
SEM images of (**a**) pure PPy (**b**) PPy/ZrO_2_ 4.

Figure 6 shows SEM images of pure PPy and a PPy/ZrO_2_4 composite. Pure PPy has a porous morphology with bigger aggregated particles and a large particle size. In the segregated form, the PPy/ZrO_2_4 composite has a granular morphology with smaller particles and reduced particle size. The ZrO_2_ particles appear to be evenly distributed over the porous surface of the PPy matrix. This causes an increase in the conductivity of the composite because of the ease of an electron hoping mechanism [36].

### 3.7. Cyclic Voltammetric (CV) Analysis

**Figure 7:**
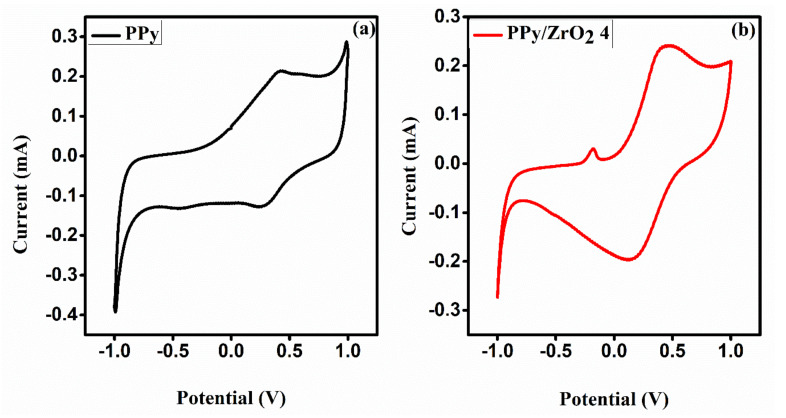
Cyclic voltammograms of (**a**) PPy and (**b**) PPy/ZrO_2_ 4 composite.

The electrochemical properties were studied by cyclic voltammetry (CV). Figure 7a,b shows the CV curves of neat PPy and tPPy/ZrO_2_4 composite, respectively. CV curves were recorded using a three-electrode setup in acidic medium containing a 1 molar H_2_SO_4_ solution as the electrolyte, with glassy carbon, gold and silver-silver chloride electrodes serving as working, counter and reference electrodes, respectively. A potential window ranging from −1 to 1 V was selected at a scan rate of 100 mV/s. PPy’s CV curves have well-defined redox peaks. At a scan rate of 100 mV/s, the PPy voltammogram reveals an anodic peak potential (Epa) of 0.419 V with current density 0.214 mA and a cathodic peak potential (Epc) of 0.280 V with current density −0.125 mA. The cyclic voltammogram of the PPy/ZrO_2_4 composite in Figure 7b shows an anodic peak potential (Epa) of 0.462 V with current density 0.241 mA and a cathodic peak potential (Epc) of 0.151 V with current density −0.196 mA. According to the CV curves, the current density has risen from 0.214 to 0.241 mA at the same scan rate (100 mV/s) and potential window, demonstrating that the composite material has a better response than the neat PPy modified electrode.

### 3.8. Effect of Scan Rate

**Figure 8:**
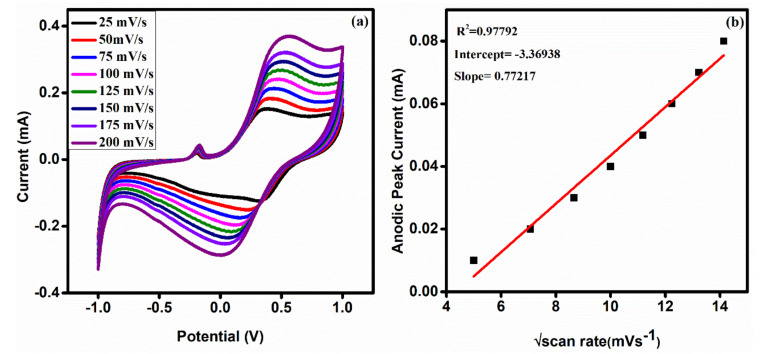
(**a**) CV of PPy/ZrO_2_ 4 at different scan rates (25–200 mV/s), and (**b**) plot of Ipa versus square root of scan rate.

Figure 8a demonstrates the influence of scan rate on the PPy/ZrO_2_ 4 modified electrode, which displays an increase in redox peaks as the scan rate increases from 25 to 200 mV/s. Figure 8b shows that the square root of scan rate and anodic peak current (Ipa) have an excellent linearity (R^2^ = 0.97792), indicating that the electron transfer reaction of the PPy/ZrO_2_ 4 modified electrode with the electrolyte solution is a diffusion controlled process.

### 3.9. Galvanostatic Charge Discharge Studies

**Figure 9:**
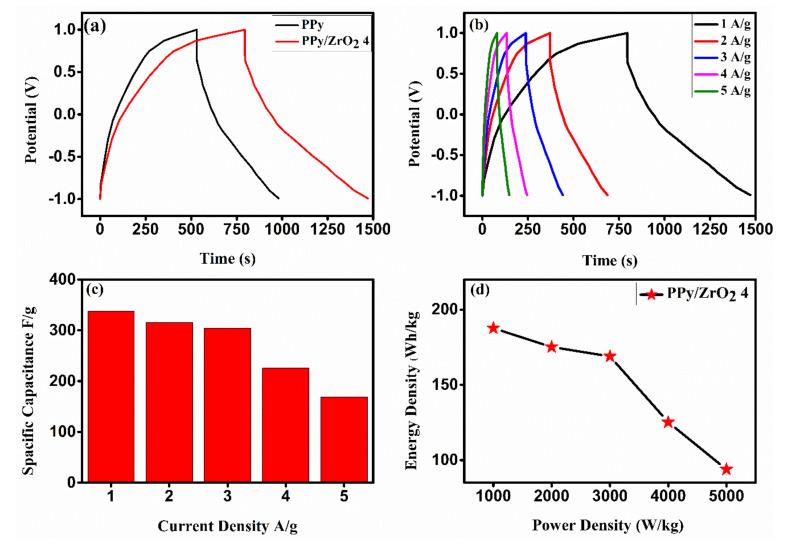

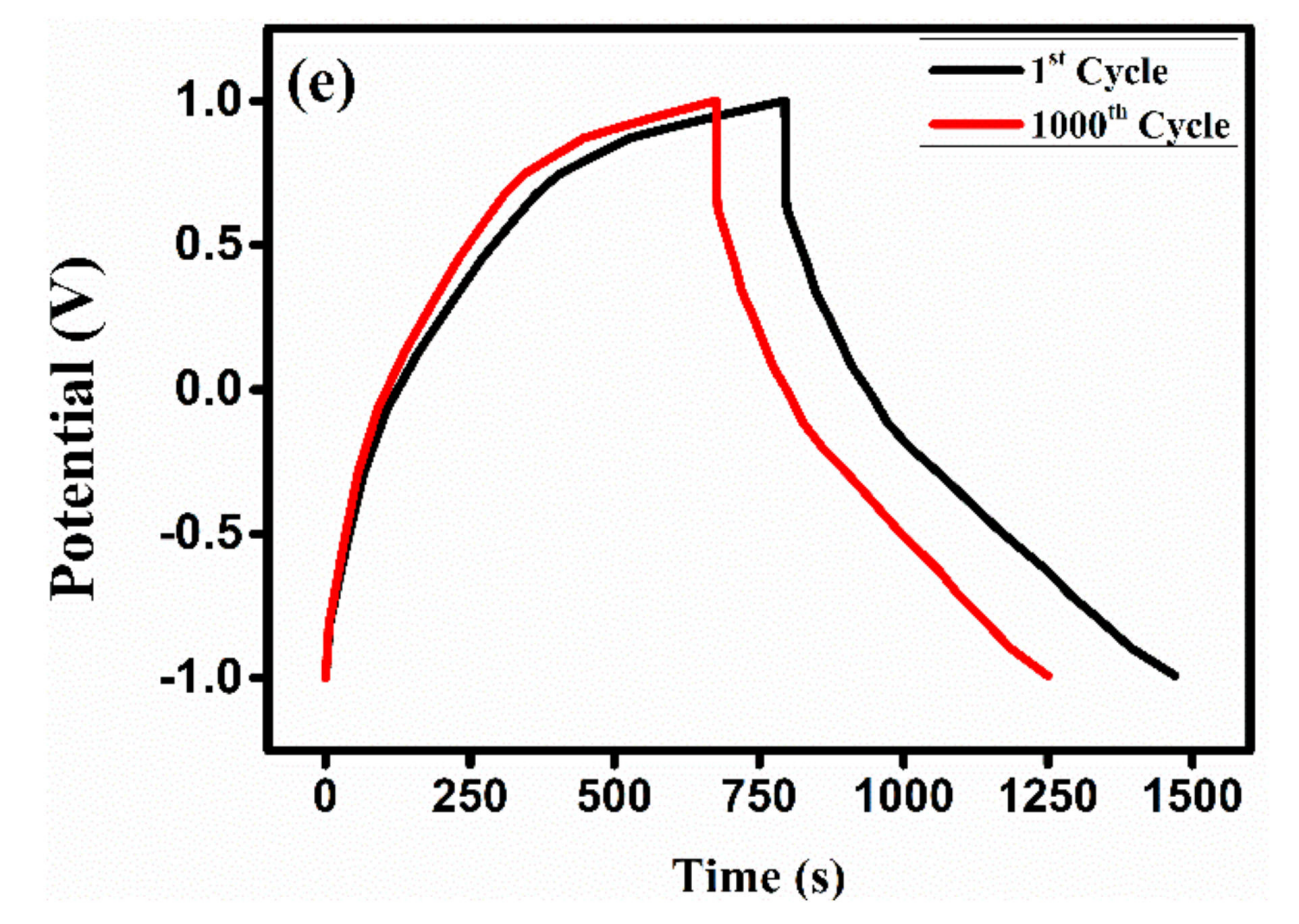
GCD curve of (**a**) PPy and PPy/ZrO_2_ 4, and (**b**) PPy/ZrO_2_ 4 at different current density. (**c**) Specific capacitance versus current density. (**d**) Ragone plot. (**e**) Comparison of GCD curves of PPy/ZrO_2_ 4, i.e., 1^st^ cycle and 1000^th^ cycle.

Figure 9a shows the galvanostatic charge discharge (GCD) of PPy and PPy/ZrO_2_ 4 composite in a potential window of −1 to 1 V in 1.0 M H_2_SO_4_ solution. The applicability of the fabricated supercapacitor electrodes was studied using various parameters such as specific capacitance (*Cs*), energy density (*E*), and power density (*P*). The specific capacitance of the fabricated supercapacitor electrodes was calculated using Equation (3).

(3) $$Cs=\frac{I\times \Delta t}{m\times \Delta V}$$

where *I* represents the current charge/discharge (A), Δ*t* is the time of discharge, Δ*V* and *m* represent the potential window and mass deposited on the GSC electrode, respectively. Equations (4) and (5) help to deduce the energy and power densities, respectively.

(4) $$E=\frac{1}{2\times 3.6}Cs\times \Delta V^{2}$$

(5) $$P=\frac{E}{\Delta t}\times 3600$$

where *Cs* represent specific capacitance, *E* and *P* symbolized energy and power densities.

As can be seen in Figure 9b, the PPy/ZrO_2_ 4 composite shows a long charge discharge time at a current density of 1 A/g. The charge/discharge time versus potential is linear in all of the curves, which is characteristic of capacitors. The specific capacitance of the PPy/ZrO_2_ composite was 337.83 F/g at a current density of 1 A/g, whereas PPy had a specific capacitance of 225.225 F/g. The high specific capacitance of the PPy/ZrO_2_ 4 electrode can be attributed to its smaller particle size and well-defined structure, which reduce ion diffusion length and increase ion and electron transport kinetics in electrodes and at the electrode/electrolyte interface [37]. Furthermore, at current densities ranging from 01 to 05 A/g, the specific capacitance of PPy/ZrO_2_ 4 was 337.83 F/g to 168.92 F/g respectively. As a result, the electrode material in a supercapacitor, PPy/ZrO_2_4, has good capacitance performance. Simultaneously, comparing GCD curves at various current densities reveals that the capacitance of sample PPy/ZrO_2_ 4 increases as the current density decreases [38]. At a higher value of current density, the ions or electrons are mainly adsorbed on the upper surface of the working electrode and hindered because of a short time at a high current density to reach the whole surface. Table 3 compares the specific capacitance and current density of the PPy/ZrO_2_ electrode used in this work to those of other well-known electrode materials, demonstrating that PPy/ZrO_2_ outperforms them.

## 4. Conclusions

We found that in-situ chemical oxidative polymerization is a simple way to successfully synthesized PPy and PPy/ZrO_2_ composites with outstanding electrochemical characteristics. The aromatic ring of the polymer exhibits π-π* transition in the UV-visible region. The presence of ZrO_2_ in the PPy matrix was verified by vibrational spectroscopy. Sharp and fine XRD spectra were detected after the inclusion of ZrO_2_ particles in PPy. The surface morphology showed a granular structure and agglomeration of particles with a higher concentration of ZrO_2_. The thermograms show an excellent thermal stability of PPy by the addition of ZrO_2_. Correspondingly, the modified electrode with the PPy/ZrO_2_ 4 composite through three electrode system showed the specific capacitance of 337.83 F/g at a current density of 1 A/g and excellent energy density of 187.67 Wh/kg and an outstanding power density of 1000 W/kg. Moreover, the modified electrode PPy/ZrO_2_ 4 demonstrated excellent specific capacitance performance and retained 85% of its specific capacitance after 1000 charge-discharge cycles. The modified PPy/ZrO_2_ 4 electrode is a promising material for making CPs supercapacitor electrodes due to its excellent electrochemical performance and cycle stability.

## Figures and Tables

**Table 1:** List of chemicals used and their molecular weight, density, and purity.

S. No.	Chemical Name	Molecular Formula	Molecular Weight	Density	% Purity
1	Pyrrole	C_4_H_4_NH	67.09 g/mol	0.966 g/mL	99.0%
2	DBSA	C_18_H_30_O_3_ S	326.49 g/mol	0.992 g/mL	70 wt. % in isopropanol
3	Ammonium persulphate	(NH4)_2_S_2_O_8_	228.20 g/mol	1.98 g/mL	98%
4	Methanol	CH_3_OH	32.04 g/mol	0.79 g/mL	98.55%
5	NMP	C_5_H_9_NO	99.13 g/mol	1.03 g/mL	99.5%
6	Chloroform	CHCl_3_	119.3 g/mol	1.492 g/mL	99.4%
7	Sulfuric acid	H_2_SO_4_	98.08 g/mol	1.84 g/mL	97%
8	Acetone	CH_3_COCH_3_	58.080 g/mol	0.7845 g/mL	99%
9	DMSO	C_2_H_6_OS	78.13 g/mol	1.01 g/mL	99%

**Table 2:** Weight loss at different temperature and weight retention.

	%Weight Loss				%Weight Retention
Sample	150 °C	200 °C	400 °C	600 °C	800 °C
PPy	4.56	6.72	27.16	54.95	21.29
PPy/ZrO_2_ 1	3.72	5.43	25.21	43.11	54.24
PPy/ZrO_2_ 2	5.04	6.63	21.37	38.83	55.07
PPy/ZrO_2_ 3	2.24	3.64	20.49	36	60.03
PPy/ZrO_2_ 4	4.92	5.84	17.55	32.76	63.27

**Table 3:** Comparative analysis of PPy/ZrO_2_ with other electrode materials.

Electrode Material	Specific Capacitance (F/g)	Current Density (A/g)	Reference
PPy/ZnO	161.02	0.5	[25]
PPy/GO	249	2 mA cm^−2^	[39]
PPy/MnO_2_	205	1	[40]
PPy/CeO_2_	193	1	[41]
PPy/β MnO_2_	297	1	[42]
PPy/ZrO_2_	337.83	1	This work

## Data Availability

Not applicable.

## References

[1] Fang S., Li J., Xiang C., Zou Y., Xu F., Sun L., Zhang J. (2020). Anchoring sea urchin-like cobalt-nickel carbonate hydroxide on 3D carbon sponge for electrochemical energy storage. J. Alloy. Compd..

[2] Cai C., Zou Y., Xiang C., Chu H., Qiu S., Sui Q., Xu F., Sun L., Shah A. (2018). Broccoli-like porous carbon nitride from ZIF-8 and melamine for high performance supercapacitors. Appl. Surf. Sci..

[3] Liu Y., Xiang C., Chu H., Qiu S., McLeod J., She Z., Xu F., Sun L., Zou Y. (2020). Binary Co–Ni oxide nanoparticle-loaded hierarchical graphitic porous carbon for high-performance supercapacitors. J. Mater. Sci. Technol..

[4] Zhang L.L., Zhao X.S. (2009). Carbon-based materials as supercapacitor electrodes. Chem. Soc. Rev..

[5] Arico A.S., Bruce P., Scrosati B., Tarascon J., van Schalkwijk W. (2011). Nanostructured materials for advanced energy conversion and storage devices. Materials for Sustainable Energy: A Collection of Peer-Reviewed Research and Review Articles from Nature Publishing Group.

[6] Meng C., Liu C., Chen L., Hu C., Fan S. (2010). Highly Flexible and All-Solid-State Paperlike Polymer Supercapacitors. Nano Lett..

[7] Kaempgen M., Chan C.K., Ma J., Cui Y., Gruner G. (2009). Printable Thin Film Supercapacitors Using Single-Walled Carbon Nanotubes. Nano Lett..

[8] Weng Z., Su Y., Wang D.-W., Li F., Du J., Cheng H.-M. (2011). Graphene-Cellulose Paper Flexible Supercapacitors. Adv. Energy Mater..

[9] Noori A., El-Kady M.F., Rahmanifar M.S., Kaner R.B., Mousavi M.F. (2019). Towards establishing standard performance metrics for batteries, supercapacitors and beyond. Chem. Soc. Rev..

[10] Woo S.-W., Dokko K., Kanamura K. (2008). Composite electrode composed of bimodal porous carbon and polypyrrole for electrochemical capacitors. J. Power Sources.

[11] Kim B., Ko J., Wallace G. (2008). A novel capacitor material based on Nafion-doped polypyrrole. J. Power Sources.

[12] Ghenaatian H., Mousavi M., Rahmanifar M. (2012). High performance hybrid supercapacitor based on two nanostructured conducting polymers: Self-doped polyaniline and polypyrrole nanofibers. Electrochim. Acta.

[13] Yuan L., Yao B., Hu B., Huo K., Chen W., Zhou J. (2013). Polypyrrole-coated paper for flexible solid-state energy storage. Energy Environ. Sci..

[14] Wang Z.-L., Guo R., Ding L.-X., Tong Y.-X., Li G.-R. (2013). Controllable Template-Assisted Electrodeposition of Single- and Multi-Walled Nanotube Arrays for Electrochemical Energy Storage. Sci. Rep..

[15] Snook G.A., Kao P., Best A.S. (2011). Conducting-polymer-based supercapacitor devices and electrodes. J. Power Sources.

[16] Chen S.S., Zhang H.R., Todd I. (2014). Phase-separation-enhanced plasticity in a Cu_47.2_Zr_46.5_Al_5.5_Nb_0.8_ bulk metallic glass. Scr. Mater..

[17] Yu M., Zeng Y., Zhang C., Lu X., Zeng C., Yao C., Yang Y., Tong Y. (2013). Titanium dioxide@polypyrrole core–shell nanowires for all solid-state flexible supercapacitors. Nanoscale.

[18] Zhang X., Zeng X., Yang M., Qi Y. (2014). Investigation of a Branchlike MoO_3_/Polypyrrole Hybrid with Enhanced Electrochemical Performance Used as an Electrode in Supercapacitors. ACS Appl. Mater. Interfaces.

[19] Gao F., Hou X., Wang A., Chu G., Wu W., Chen J., Zou H. (2016). Preparation of polypyrrole/TiO_2_ nanocomposites with enhanced photocatalytic performance. Particuology.

[20] Jadhav N., Kasisomayajula S., Gelling V.J. (2020). Polypyrrole/Metal Oxides-Based Composites/Nanocomposites for Corrosion Protection. Front. Mater..

[21] Grari O., Taouil A.E., Dhouibi L., Buron C., Lallemand F. (2015). Multilayered polypyrrole–SiO_2_ composite coatings for functionalization of stainless steel: Characterization and corrosion protection behavior. Prog. Org. Coat..

[22] Dey S., Kar A.K. (2021). Effect of Forster resonance energy transfer on the photoluminescence of PPy-ZnO composite. J. Sol.-Gel. Sci. Technol..

[23] Chigondo M., Chigondo F., Nyamunda B. (2021). Synthesis of hydrous CeO_2_ polypyrrole nanocomposite as a rapid and efficient adsorbent for defluoridation of drinking water. Environ. Nanotechnol. Monit. Manag..

[24] Dakshayini B., Reddy K.R., Mishra A., Shetti N.P., Malode S.J., Basu S., Naveen S., Raghu A.V. (2019). Role of conducting polymer and metal oxide-based hybrids for applications in ampereometric sensors and biosensors. Microchem. J..

[25] Xue J., Yang Q., Guan R., Shen Q., Liu X., Jia H., Li Q. (2020). High-performance ordered porous Polypyrrole/ZnO films with improved specific capacitance for supercapacitors. Mater. Chem. Phys..

[26] Kim M.S., Park J.H. (2011). Polypyrrole/titanium oxide nanotube arrays composites as an active material for supercapacitors. J. Nanosci. Nanotechnol..

[27] Yang H., Zhou K., Pan D., Liu X., Yang M., Zhu X.J. (2018). Polypyrrole@silica composites as high performance electrode materials for Lithium-ion batteries. Mater. Sci. Mater. Electron..

[28] Bekhoukh A., Moulefera I., Sabantina L., Benyoucef A. (2021). Development, Investigation, and Comparative Study of the Effects of Various Metal Oxides on Optical Electrochemical Properties Using a Doped PANI Matrix. Polymers.

[29] Habelhames F., Nessark B., Bouhafs D., Cheriet A., Derbal H. (2010). Synthesis and characterisation of polypyrrole–indium phosphide composite film. Ionics.

[30] Vasilyeva S.V., Vorotyntsev M.A., Bezverkhyy I., Lesniewska E., Heintz O., Chassagnon R. (2008). Synthesis and Characterization of Palladium Nanoparticle/Polypyrrole Composites. J. Phys. Chem. C.

[31] Bilal S., Perveen F., Shah A. (2015). Chemical synthesis of polypyrrole doped with dodecyl benzene sulfonic acid. J. SciInnov Res..

[32] Irfan M., Shakoor A., Majid A., Hassam N., Ahmed N. (2019). Study of Structural, Thermal and Dielectric Modulus of PPy–DBSA–Zirconium Oxide Composites. Russ. J. Phys. Chem. B.

[33] Wang J., Yin W., He X., Wang Q., Guo M., Chen S. (2016). Good Biocompatibility and Sintering Properties of Zirconia Nanoparticles Synthesized via Vapor-phase Hydrolysis. Sci. Rep..

[34] Sultan A., Ahmad S., Mohammad F. (2017). Synthesis, Characterization and Electrical Properties of Polypyrrole/ Zirconia Nanocomposite and its Application as Ethene Gas Sensor. Polym. Polym. Compos..

[35] Yamani K., Berenguer R., Benyoucef A., Morallón E. (2019). Preparation of polypyrrole (PPy)-derived polymer/ZrO_2_ nanocomposites. J. Therm. Anal. Calorim..

[36] Jayamurgan P., Ponnuswamy V., Ashokan S., Mahalingam T. (2013). The effect of dopant on structural, thermal and morphological properties of DBSA-doped polypyrrole. Iran. Polym. J..

[37] Wu W., Yang L., Chen S., Shao Y., Jing L., Zhao G., Wei H. (2015). Core–shell nanospherical polypyrrole/graphene oxide composites for high performance supercapacitors. RSC Adv..

[38] Liu X., Yang J., Li X., Li Q., Xia Y. (2020). Fabrication of polypyrrole (PPy) nanotube electrode for supercapacitors with enhanced electrochemical performance. J. Mater. Sci. Mater. Electron..

[39] Wang P., Zheng Y., Li B. (2013). Preparation and electrochemical properties of polypyrrole/graphite oxide composites with various feed ratios of pyrrole to graphite oxide. Synth. Met..

[40] Yuan L., Wan C., Zhao L. (2015). Facial in-situ synthesis of MnO_2_/PPy composite for supercapacitor. Int. J. Electrochem. Sci..

[41] Wang X., Wang T., Liu D., Guo J., Liu P. (2016). Synthesis and Electrochemical Performance of CeO_2_/PPy Nanocomposites: Interfacial Effect. Ind. Eng. Chem. Res..

[42] Zang J., Li X. (2011). In situ synthesis of ultrafine β-MnO_2_/polypyrrole nanorod composites for high-performance supercapacitors. J. Mater. Chem..

